# Dose caffeinated energy drink is a consideration issue for endurance performance

**DOI:** 10.3389/fphys.2022.999811

**Published:** 2022-10-28

**Authors:** Jie-Ping Wang, Chen-Chan Wei, Yun-Dong Peng, Hsuan-Yun Wang, Chi-Hsiang Hung, Yin-Hui Hong, Yuh-Feng Liou, Chien-Wen Hou

**Affiliations:** ^1^ Laboratory of Exercise Biochemistry, Institute of Sports Sciences, University of Taipei, Taipei, Taiwan; ^2^ Department of Aquatic Sports, University of Taipei, Taipei, Taiwan; ^3^ School of Sport Medicine and Rehabilitation, Beijing Sport University, Beijing, China; ^4^ Department of Physical Education, Shih Hsin University, Taipei, Taiwan; ^5^ Department of Ball Sports, University of Taipei, Taipei, Taiwan; ^6^ Department of Psychology and Counseling, University of Taipei, Taipei, Taiwan; ^7^ General Education Center, University of Taipei, Taipei, Taiwan

**Keywords:** anserine, elderberry, haematology, oxidative stress, sprint triathlon

## Abstract

Caffeinated energy drinks are commonly taken to improve exercise performance, but there are few studies on the influence of different doses on an athlete’s performance. We conducted a double-blind, randomized, counter-balanced, and crossover research study to examine the effects of low caffeinated energy drink (Low ED) or high caffeinated energy drink (High ED) supplement on the performance, haematological response, and oxidative stress in triathletes. Twelve male participants underwent three testing sessions separated by weekly intervals, consisting of sprint triathlon training (0.75 km swim, 20 km cycle, and 5 km run). Before and during the trials, participants were randomly provided with either placebo (PLA) group, Low ED group, or High ED group. Exercise performance in the High ED group decreased significantly compared with the PLA and Low ED groups (*p* < 0.05). However, participants in the Low ED group also experienced an improved performance (*p* = 0.054). Analysis of variance revealed no differences among the three groups in cortisol and testosterone levels, or the Borg Rating of Perceived Exertion score (*p* > 0.5). Furthermore, superoxide dismutase (SOD) was reduced with exercise and were lowest in the High ED group. However, compared with PLA, a significant decrease of thiobarbituric acid reactive substances (TBARS) was observed in Low ED and High ED groups (*p* < 0.05). This indicates that caffeinated energy drink consumption may improve performance and reduce oxidative stress in sprint triathlon athletes. However, individual differences should be considered when supplementing with caffeinated energy drinks to decrease side effects.

## Introduction

A triathlon, which combines phases of swimming, cycling, and running, is one of the main long-distance races in the world. The full distance is the most famous triathlon. However, shorter triathlon competitions, such as the Olympic triathlon or sprint triathlon, have become more popular because it allows amateur and recreational athletes to participate (Jeukendrup et al., 2005). It is a strenuous competition that requires high energy expenditure and generates high physical and metabolic stress (Knez et al., 2007). Therefore, several athletes take energy drink supplements during training (Potgieter et al., 2018). However, the effect for sprint triathlon is still not clearly.

Energy drinks are commonly taken to improve exercise performance not only among professional athletes, but also among other sports people (Hoffman et al., 2007; Chester and Wojek, 2008; Petróczi et al., 2008). Depending on the composition of the energy drinks, some are more commonly used by endurance athletes, while others are more suitable for strength athletes. The most common supplement is caffeine. Caffeine is an effective ergogenic agent, which can delay the time to fatigue during endurance sports (Southward et al., 2018; Shen et al., 2019). Many studies have shown that caffeine affects endurance performance but has no effect on anaerobic performance (Hoffman et al., 2007; Hogervorst et al., 2008; Woolf et al., 2009; Southward et al., 2018). A study showed that 89% of the athletes participating in the Triathlon World Championships admitted that they planned to use caffeine supplements before the competition (Desbrow and Leveritt, 2006). Caffeine supplementation has been shown to improve the swimming performance of triathlon athletes during competition (Potgieter et al., 2018).

The triathlon combines periods of prolonged exercise and high intensity which results in muscle fatigue and damage (Bertola et al., 2014). Exercise has been reported to result in leucocytosis (Peake et al., 2017), and for caffeinated energy drink consumption seem to have further augmented these responses (Phillips et al., 2014; Stopa et al., 2020). Testosterone and cortisol are indicators of anabolism and catabolism in endurance exercise, are usually measured to assess the stress imposed by exercise (Anderson et al., 2016; Vaamonde et al., 2021). Exercise-induced oxidative stress increases muscle fatigue and damages muscle function (Koechlin et al., 2004; Aguiar et al., 2008). Under aerobic endurance stress, the reactive radical oxygen species generation increases. Oxidative stress starts when the antioxidant system does not adapt to the excessive production of reactive radical oxygen species (Ji, 1999). The enzymes such as superoxide dismutase (SOD), catalase (CAT), and glutathione peroxidase (GSH-Px), and non-enzymatic substances such as reduced glutathione (GSH), which can prevent exercise-induced oxidative stress (Pinho et al., 2006). It is important to identify approaches that reduce exercise-induced oxidative stress and help improve exercise performance. Elderberry, anserine and caffeine may have an antioxidant effect and protect cells from oxidative damage (Abdel-Hady et al., 2015; Alkhatib et al., 2020; Liu et al., 2022).

Furthermore, most studies only discuss the influence of the same doses of caffeine on an athlete’s performance (Ganio et al., 2009; Southward et al., 2018; Shen et al., 2019). However, the effects of caffeine on the human body vary drastically between individuals (Turley and Gerst, 2006; Nehlig, 2018; Potgieter et al., 2018; Durkalec-Michalski et al., 2019). Therefore, the purpose of this study was to examine the effects of varied doses of caffeinated energy drink supplementation on oxidative stress and performance in sprint triathletes. We make a hypothesis that it would be distinct in different dosage of caffeinated energy drink.

## Materials and methods

### Participants

Twelve male triathletes (height, 171.8 ± 1.5 cm; weight, 63.5 ± 2.3 kg; age, 20.8 ± 0.4 years) all voluntarily agreed to take the digitally provided, anonymous, online survey to enrol in this study. The inclusion criteria required that participants were healthy male collegiate triathletes. The exclusion criteria were: (Jeukendrup et al., 2005): taking any medicines; (Knez et al., 2007) any health problems that may have been exacerbated by caffeine; (Potgieter et al., 2018) habitual caffeine consume The procedures and purpose of the study, including the right to freely withdraw, were explained to the participants and their informed consent was obtained. This study was approved by Institutional Review Board of Taipei University (IRB-2021-021). All participants were adults who matched the requirements for this research and had no health or drug related problems.

### Experimental design

This study followed a double-blind, randomized, counter-balanced, and crossover design. Three identical sprint triathlon tests (0.75 km swim, 20 km cycle, and 5 km run) were performed. Swimming is in indoor 50 m swimming pool, cycling is on stationary bicycle, and running in outdoor 400 m field. Experimental trials began at the same time for 3 days, with each testing session separated by 1 week. The weather conditions of the three experimental days are similar.

Participants were randomly assigned to a placebo (PLA) group, low-dosage caffeinated energy drink (Low ED) group, or high-dosage caffeinated energy drink (High ED) group by an external researcher. Supplement is given to the participants in an opaque cup by another researcher. Participants were provided the caffeinated energy drink supplement or PLA 1 h before the test and during transitions (swim → cycle). Participants began warm-ups 30 min before the test.

### Supplement intervention

Each participant ingested either caffeinated energy drink supplement or PLA. The supplement is commercially marked as Energy Drink (Power Probiotics, Taiwan). Each supplement consisted of 300 ml water mixed with 3 g of the Energy Drink powder, which contained 111 mg of caffeine and 674 mg of another energy matrix (144 mg anserine, 200 mg of elderberry, and 330 mg vitamins-minerals mixed). It contains 8.6 kilocalorie (kcal) of energy. PLA was the same volume of purple grape juice drink. Both drinks were matched to be similar in taste and appearance.

**FIGURE 1:**
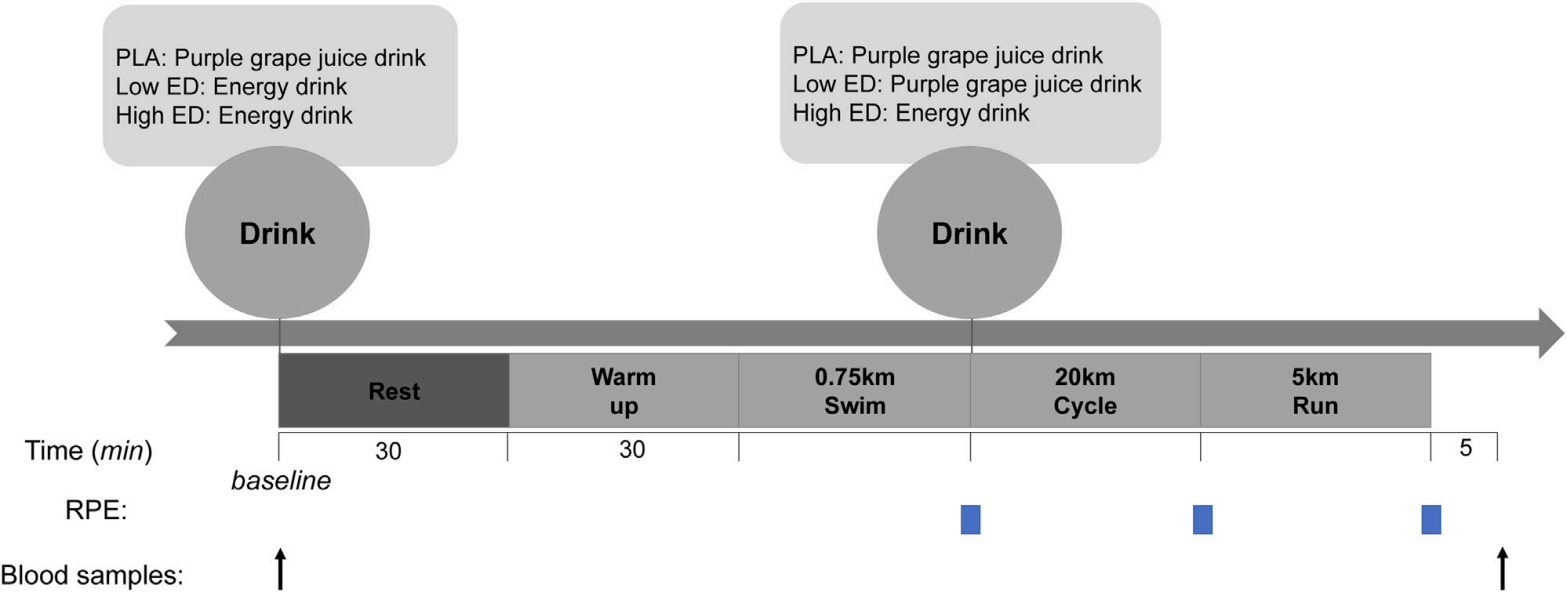
Study protocol and interventions.

In the PLA group, participants ingested the PLA at baseline and during transition between tests (from swimming to cycling). The Low ED group ingested the caffeinated energy drink supplement at baseline and ingested PLA during the transitions. The High ED group ingested the caffeinated energy drink supplement at baseline and again during transitions (Figure 1). The dosage of High ED is twice than the Low ED. The caffeine dosage in Low ED is 111 mg, and in High ED is 222 mg. There are 8.6 kcal in Low ED, and for High ED is 17.2 kcal.

### Dietary and exercise standardisation

Meals and training were selected individually by the participants as usual, but were approved by the researchers. Participants were instructed to eat the same diet provided 2 h before each trial, and were asked to disclose the use of any performance-enhancing supplements or drugs. Only participants who were not using any ergogenic supplements were included in the study. In order to exclude confounding effects of caffeine withdrawal symptoms, participants were required to abstain from caffeine or caffeine-containing products, and to not perform any strenuous exercise 48 h prior to the experiment.

### Sample and data collection

On the experiment day, participants finished their lunch 2 h before arrival. Blood samples were collected by registered phlebotomists before baseline and 5 min after finishing the exercise. Times to complete all race stages were recorded. The Borg Rating of Perceived Exertion (RPE, 1–10 scale) was used to determine the immediate RPE score during sprint triathlon.

To obtain plasma or serum, the blood samples with 5 ml collected into blood sampling tubes with or without ethylene diamine tetraacetic acid (EDTA), respectively, were centrifuged at 3000 rpm at 4°C for 10 min and then stored at −80°C until analysis. Full blood counts were obtained using automated haemocytometry (UniCel DxH900, Beckman Coulter^®^, America). Thiobarbituric acid reactive substances (TBARS), SOD, and CAT were measured using an enzyme-linked immunosorbent assay (ELISA) kit (Cayman Chemical, Ann Arbor, MI, United States); cortisol and testosterone levels were also measured using an ELISA kit (IBL^®^, Minneapolis, MN, America). GSH and oxidized glutathione (GSSG) were estimated using Glutathione fluorometric assay kit (BioVision, Milpitas, CA, United States) and an ELISA plate reader (Infinite M200Pro, Tecan Group Ltd., Mannedorf, Switzerland).

### Statistical analysis

The evaluations were analysed using the one-way analysis of variance and repeated measurement tests. In the event of a significant main effect, post-hoc comparisons were conducted using the Tukey’s test. Statistical significance was set at *p* < 0.05. Data are expressed as mean ± standard error. Effect sizes (Cohen’s d) were reported where appropriate. Parametric effect sizes were defined as large (d > 0.8), moderate (0.5–0.8), and small (<0.5) (Cohen, 2013).

## Results

**FIGURE 2:**
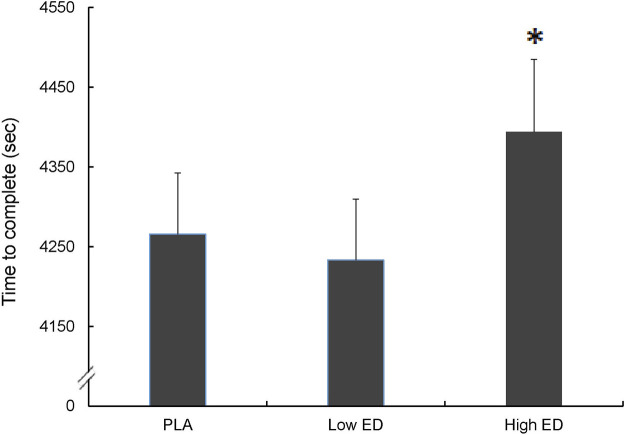
Sprint triathlon performance with placebo (PLA), low-dosage energy drink (Low ED) group and high-dosage energy drink (High ED) group. *Significant difference compare with PLA and Low ED (*p* < 0.05). Values are expressed as mean ± SE.

Completion times were significantly increased in the High ED group compared with the PLA and Low ED groups, which indicated that the exercise performance decreased significantly. (4394 ± 315 vs. 4266 ± 266 s, *p* = 0.03; 4394 ± 315 vs. 4233 ± 26 s, *p* = 0.03). There was no difference in completion times between the PLA and Low ED supplementation groups (Figure 2).

**FIGURE 3:**
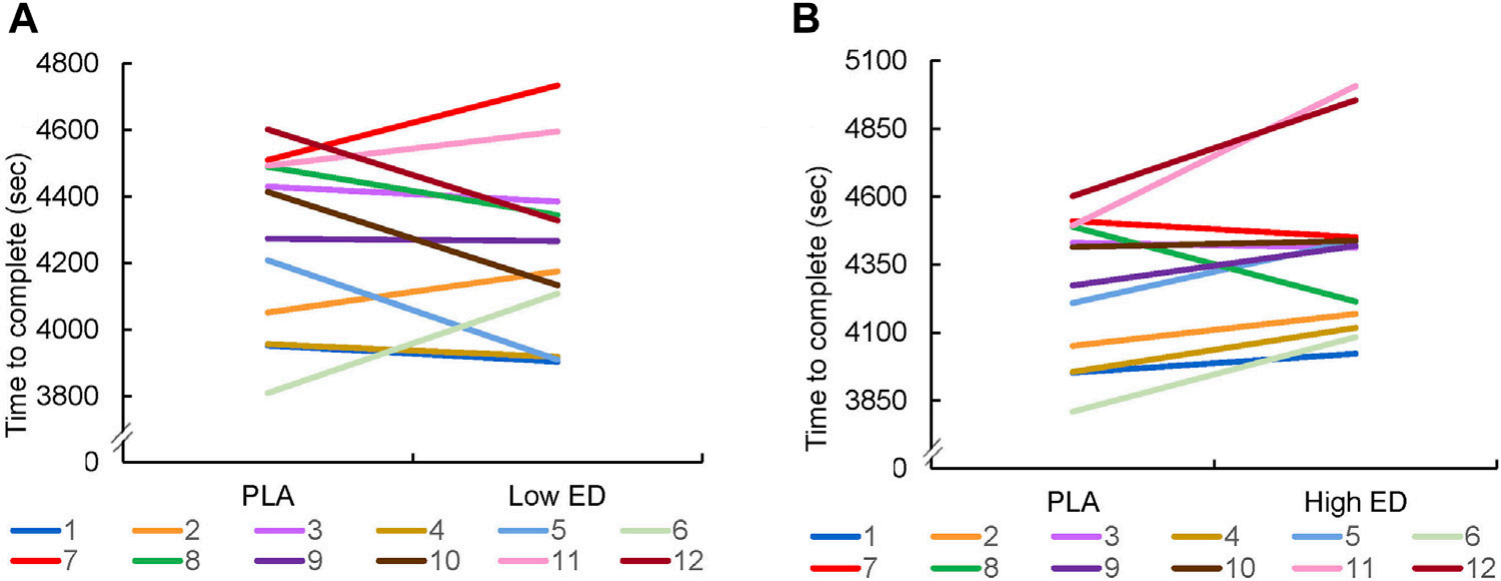
Individual sprint triathlon performance with placebo (PLA) vs. low-dosage energy drink (Low ED) group **(A)** and placebo (PLA) vs. high-dosage energy drink (High ED) group **(B)**. Note that y-axes differ, as they were adapted for best spread of data.

Figure 3 shows the total time of individual performances by group of different caffeinated energy drink dosages. Low ED supplementation was associated with triathlon performance improvements in 75% (9/12) of athletes; however, only 25% (3/12) of athletes in the High ED supplementation group saw improvement in their performance. Compared with other studies, in relation to the demonstrated ergogenic effect, the performance of most athletes improved after ingesting the caffeinated energy drink supplement.

**FIGURE 4:**
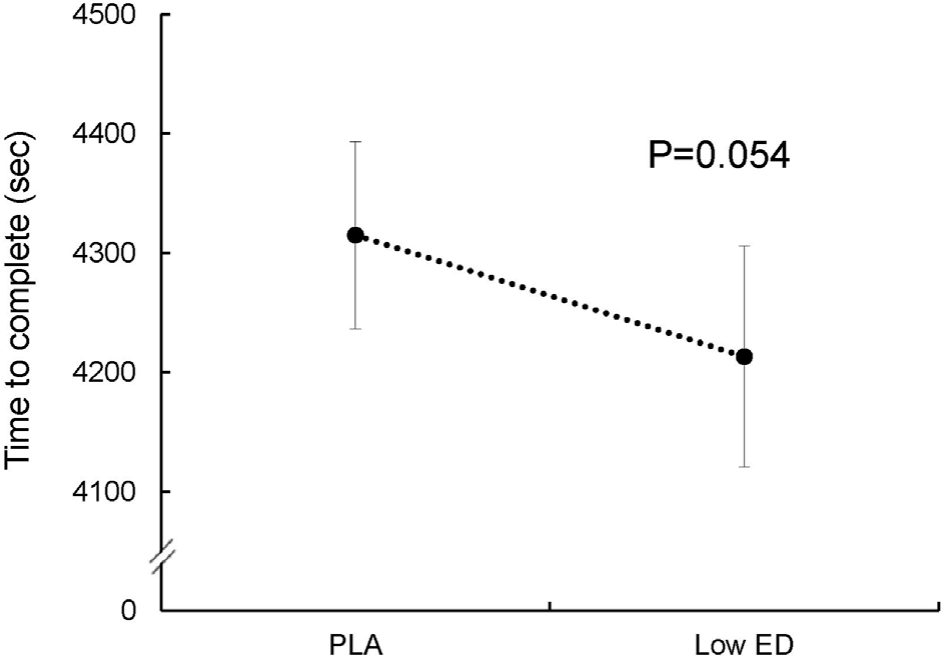
Sprint triathlon performance for nine subjects with placebo (PLA) and low-dosage energy drink (Low ED) group. (Except three subjects who may overdose). Values are expressed as mean ± SE.

In a subsequent analysis, we focused on the participants in the Low ED group who may not have experienced an overdosage of components in the caffeinated energy drink supplementation. These nine participants were compared with the PLA groups. The performance of these athletes seems to show significant improvement with Low ED group, compared with the PLA group (*p* = 0.054; effect size = 0.4) (Figure 4).

**FIGURE 5:**
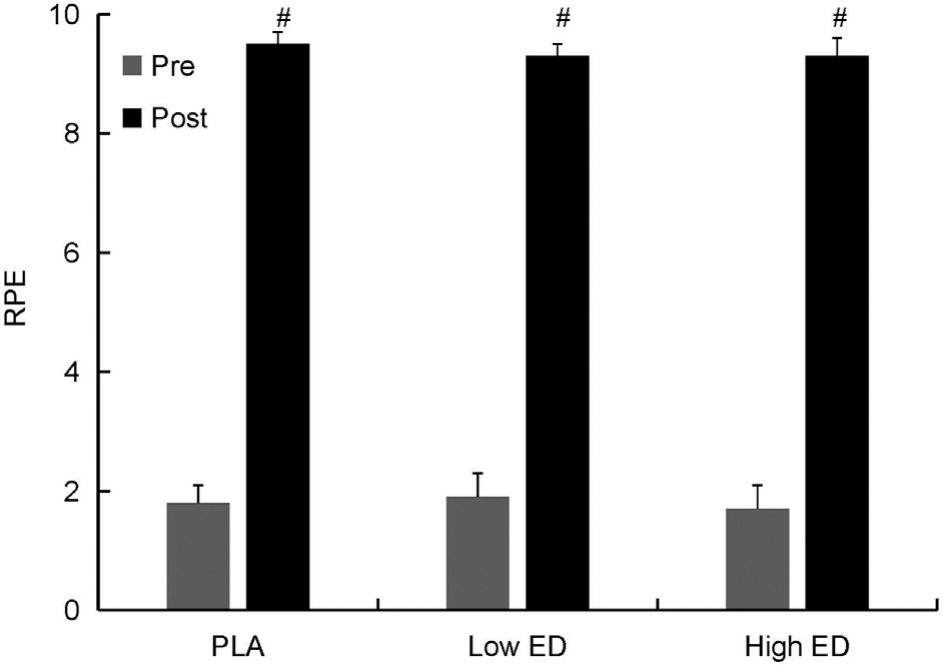
RPE before and after sprint triathlon with placebo (PLA), low-dosage energy drink (Low ED) group and high-dosage energy drink (High ED) group. Values are expressed as mean ± SE. #Significant difference compare with pre and post (*p* < 0.05). Values are expressed as mean ± SE.

**FIGURE 6:**
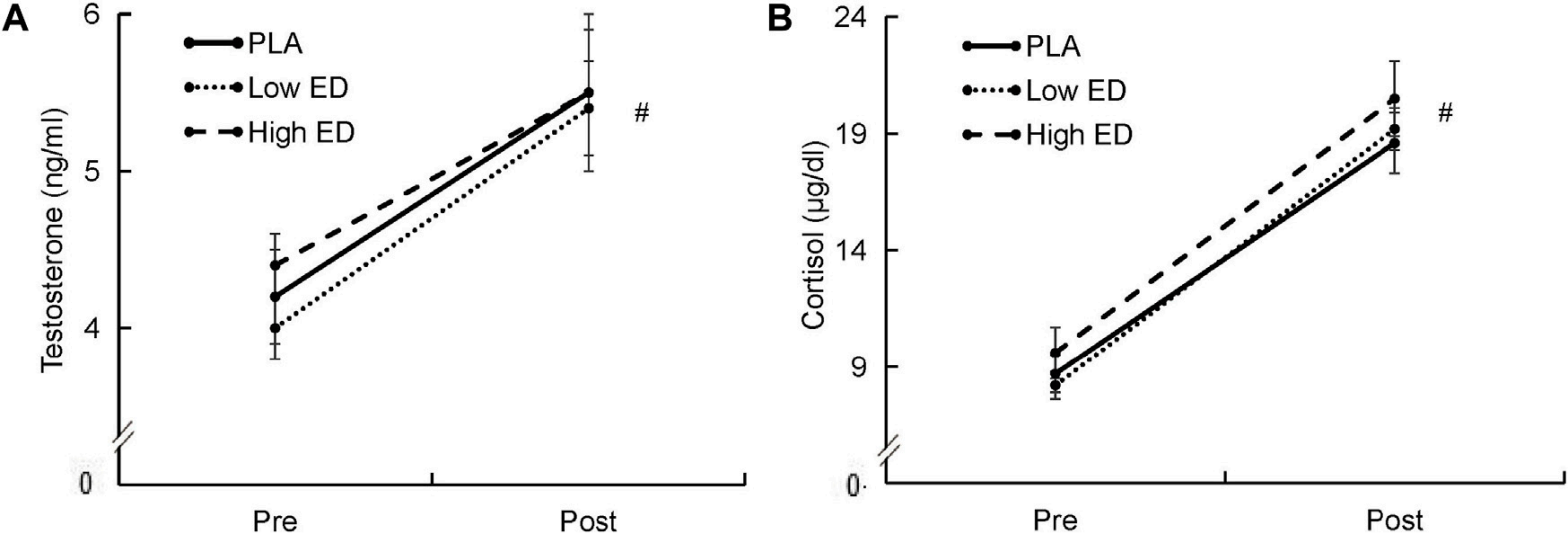
Serum testosterone **(A)** and cortisol **(B)** in triathlon with placebo (PLA), low-dosage energy drink (Low ED) group and high-dosage energy drink (High ED) group at baseline and immediately after sprint triathlon Values are expressed as mean ± SE. *Significant difference compare with pre and post (*p* < 0.05) in PLA, Low ED and High ED group. Values are expressed as mean ± SE.

The RPE of the athletes increased after exercise in all groups, but thereafter remained similar at all time points in all groups (Figure 5). Exercise resulted in significant increase of testosterone and cortisol (*p* < 0.05), however, there was no significant difference in cortisol or testosterone levels in the PLA group versus the Low ED and High ED groups (Figure 6). Exercise caused an elevation in white blood cells, specifically the neutrophils; the elevation was greater in the High ED group as compared to the other two groups. After exercise, mononuclear cell level and red blood cell count increased in all groups, bud the increase in red blood cell count in caffeinated energy drink consumption group was lesser than in the PLA group (Table 1).

**TABLE 1:** Hematological parameters with placebo (PLA), low-dosage caffeinated energy drink (Low ED) group and high-dosage caffeinated energy drink (High ED) group before and after participation in a sprint triathlon. *Significant difference at *p* < 0.05.

	PLA	Low ED	High ED	Main effect		*p* vs. L	*p* vs. H	L vs. H
	Mean ± SE			F	*p*	*p*-value (Cohen’s d)		
	White blood cell (10^3^cells/μl)							
Pre	5.9 ± 0.3	5.9 ± 0.4	5.9 ± 0.3	0.0	1.00			
Post	10.7 ± 0.5	11.8 ± 0.6	13.3 ± 1.1	3.8	0.04*	*p =* 0.11	*p =* 0.02*(0.6)	*p =* 0.07
△WBC	4.8 ± 0.6	5.9 ± 0.5	7.4 ± 1.1	4.0	0.03*	*p =* 0.09	*p =* 0.02*(0.9)	*p =* 0.06
	Neutrophils(%)							
Pre	60.0 ± 2.1	60.1 ± 2.2	59.2 ± 1.9	0.1	0.91			
Post	70.3 ± 2.2	73.4 ± 2.5	74.5 ± 3.2	1.8	0.19			
△NEU	10.2 ± 1.9	13.2 ± 2.5	15.3 ± 3.2	0.9	0.41			
	Lymphocytes(%)							
Pre	27.5 ± 1.6	29.1 ± 2.0	28.6 ± 1.4	0.5	0.63			
Post	20.8 ± 1.8	19.0 ± 2.0	18.0 ± 2.4	1.12	0.34			
△LYM	-6.7 ± 1.8	-10.1 ± 2.5	-10.7 ± 2.9	1.0	0.39			
	MONO(%)							
Pre	7.9 ± 0.7	7.1 ± 0.8	8.0 ± 0.8	0.5	0.61			
Post	6.1 ± 0.4	5.5 ± 0.7	5.1 ± 0.7	1.6	0.23			
△MONO	-1.8 ± 0.6	-1.6 ± 0.4	-2.8 ± 0.5	1.7	0.20			
	Red Blood Cell(10^6^cells/μl)							
Pre	5.1 ± 0.1	5.1 ± 0.1	5.1 ± 0.1	1.3	0.30			
Post	5.4 ± 0.1	5.3 ± 0.1	5.4 ± 0.1	0.2	0.84			
△RBC	0.3 ± 0.04	0.2 ± 0.04	0.2 ± 0.03	2.6	0.10			

Regarding oxidative stress (Table 2), CAT levels were similar in all groups. SOD levels were reduced with exercise and were lowest in athletes in the High ED group. TBARS levels increased after exercise in all groups; however, caffeinated energy drink supplementation caused a significant decrease in TBARS levels, irrespective of dosage. There were no significant differences in GSH, GSSG, or GSH/GSSG levels among the PLA group and Low or High ED groups.

**TABLE 2:** Oxidation stress response with placebo (PLA), low-dosage caffeinated energy drink (Low ED) group and high-dosage caffeinated energy drink (High ED) group before and after participation in a sprint triathlon. *Significant difference at *p* < 0.05.

	PLA	Low ED	High ED	Main effect		*p* vs. L	*p* vs. H	L vs. H
	Mean ± SE			F	*p*	*p*-value (Cohen’s d)		
	SOD (U/ml)							
Pre	2.1 ± 0.3	2.1 ± 0.3	2.1 ± 0.3	0.0	0.96			
Post	1.8 ± 0.2	1.7 ± 0.3	1.6 ± 0.3	4.1	0.03*	*p =* 0.12	*p =* 0.02*(0.2)	*p =* 0.02*(0.2)
*p*-value	*p =* 0.01*	*p =* 0.01*	*p =* 0.00*					
	Catalase (nmol/min/ml)							
Pre	46.8 ± 3.8	44.8 ± 1.8	49.0 ± 7.3	0.19	0.83			
Post	68.6 ± 4.2	63.5 ± 3.9	63.7 ± 2.7	1.7	0.20			
*p*-value	*p =* 0.00*	*p =* 0.00*	*p =* 0.03*					
	TBARS (μM)							
Pre	7.2 ± 1.6	10.2 ± 1.6	13.0 ± 3.8	1.5	0.25			
Post	13.2 ± 4	5.9 ± 0.6	6.0 ± 0.8	3.8	0.04*	*p =* 0.04*(0.8)	*p =* 0.03*(0.7)	*p =* 0.46
*p*-value	*p =* 0.07	*p =* 0.02*	*p* = 0.03*					
	GSH (μg/ml)							
Pre	11.8 ± 1.4	12.1 ± 1.1	13.2 ± 1.7	0.44	0.65			
Post	13.4 ± 1.6	13.8 ± 1.9	13.0 ± 2.3	0.14	0.87			
*p*-value	*p =* 0.19	*p =* 0.13	*p =* 0.45					
	GSSG (μg/ml)							
Pre	181.3 ± 20.3	194.3 ± 13.4	184.1 ± 10.5	0.3	0.76			
Post	184.5 ± 18.7	183.1 ± 12.2	186.5 ± 13.1	0.0	0.98			
*p*-value	*p =* 0.43	*p =* 0.20	*p =* 0.44					
	GSH/GSSG(μg/ml)							
Pre	0.08 ± 0.02	0.06 ± 0.01	0.07 ± 0.01	0.7	0.50			
Post	0.08 ± 0.01	0.08 ± 0.01	0.07 ± 0.01	0.3	0.76			
*p*-value	*p =* 0.45	*p =* 0.10	*p =* 0.48					

## Discussion

The main finding of the study was that in comparison to the placebo and Low ED groups, the High ED group showed significant increase in completion times. In the Low ED group, 75% of athletes had improved performance, whereas in the High ED group 75% of athletes showed a lower performance. The most commonly used ingredient in energy drinks is caffeine. The ergogenic effect of caffeine on endurance exercise has been extensively demonstrated (Bell et al., 1998; Bridge and Jones, 2006; Desbrow and Leveritt, 2006; Meeusen et al., 2013; Glaister et al., 2016; Christensen et al., 2017; Potgieter et al., 2018). However, excessive intake of caffeine may reduce performance (Mora-Rodriguez and Pallarés, 2014).

In a descriptive cross-sectional study, Desbrow and Leveritt found that intake of an average caffeine dose of 3.8 ± 3 mg/kg by an athlete during a triathlon had very minor side effects (Desbrow and Leveritt, 2007). Another study showed that the ergogenic effects of caffeine ingestion on neuromuscular performance differ when ingestion is at different time points. Caffeine intake in the afternoon was not only reported to have little effect on neuromuscular performance, but also increased the rate of negative side-effects (Mora-Rodríguez et al., 2015).

The International Society of Sports Nutrition summarized that caffeine is effective for enhancing sports performance in trained athletes, when consumed in low-to-moderate dosages (3–6 mg/kg) (Campbell et al., 2013). Other studies have shown similar results (Potgieter et al., 2018; Durkalec-Michalski et al., 2019). In our study, the average body weight of the participants was 63.5 ± 2.3 kg, and the athletes from the High ED group consumed 222 mg of caffeine. Although this seems to be a lower dose than reported, it still caused an overdose of caffeine in many of the athletes. According to Figures 3, 4, we suggest that the remaining participants, who showed no improvement, may also have experienced an overdosage of components in the caffeinated energy drink supplementation. A similar dose of caffeine was taken in another study on energy drinks, and the cycling performance also did not improve (Phillips et al., 2014). However, only a single dose was taken in these studies, they cannot consider whether it was caused by excessive caffeine.

The reason for this difference from other researches in results may involve inter-individual and ethnic differences in caffeine metabolism. The participants in our study are from Asia, which may be one of the reasons why the performance of some athletes in this study deteriorated or did not improve. CYP1A2 is responsible for over 90% of caffeine clearance. The large interindividual variability in the activity of CYP1A2 influences the disposition of caffeine. The activity of CYP1A2 in the South Asian population is especially lower than that of other people (Nehlig, 2018). A meta-analysis demonstrated that people possessing a fast caffeine metabolism could tolerate a higher coffee intake, especially in males, younger age groups, and individuals of Caucasian ethnicity (Denden et al., 2016). It also showed that mainly the cytochrome P450 family 1, subfamily A1-A2 (CYP1A1-CYP1A2) has been implicated in caffeine metabolism. This suggests that for some people even low doses of caffeine may lead to side effects. In addition to, gene types can also lead to different side effects (Salinero et al., 2017; Grgic et al., 2020). Therefore, individual differences should be considered when consuming caffeinated energy drinks containing caffeine in future studies.

Except for caffeine in energy drinks, for other substances, until now, almost no study shows that other energy matrix (674 mg of anserine, elderberry, vitamins, and minerals) in the caffeinated energy drink we used can cause side effects. And there is a big difference in caffeine content but little difference energy between the two group. Therefore, we infer that excessive caffeine may be the reason why some athletes’ performance did not improve. However, because our study lacked a caffeine control group, we cannot be sure whether the reduced performance observed were caused by excessive caffeine. In addition to, our participants are only males. These are research limitation of our study.

Exercise increases red and white cell counts and induces delayed-onset leukocytosis due to neutrophilia (Wang et al., 2003). Exercise-induced neutrophil mobilization and activation might be associated with muscle damage (Kawamura et al., 2018). We found a greater increase in leukocyte levels in the High ED group than in the PLA group, especially for neutrophils. This may be due to the synergic effect of caffeine (Bassini-Cameron et al., 2007), caffeine-induced increase in adrenaline could be responsible for the higher increase in neutrophil (Tauler et al., 2013).

However, another research study suggested that caffeine can affect the bone marrow directly or through the central nervous system, causing an increased release of neutrophils (Ramanaviciene et al., 2004). Their results showed that caffeine may enhance the mobilization and activation of neutrophils during exercise.

In our study, testosterone levels in the athletes did not differ among the PLA group and the Low and High ED groups. Inconsistent with other research studies (Potgieter et al., 2018), our data showed that testosterone levels increased after the sprint distance triathlon. This may be due to measurement of values at different completion times. An earlier study found an increase in serum testosterone during the first 3 h of exercise and a decrease thereafter, regardless of the type of exercise (running or walking) (Guglielmini et al., 1984). Another study evaluated two types of athletes (middle-distance and prolonged marathon runners) during an exercise duration of 40 min, and found a progressive increase in testosterone levels during the final phases of a prolonged marathon (Vuorimaa et al., 2008). This phenomenon was also corroborated by a steady increase during the last two phases of the Ironman (cycling and running) (Vaamonde et al., 2021). Our findings are similar to most studies in the field (Elloumi et al., 2008; Balthazar et al., 2012), which have demonstrated an increase in cortisol secretion after exercise, and no differences between the group that consumed the caffeinated energy drink supplement and the PLA group.

The present study also reported a significant effect on oxidation stress on sprint triathlon after consumption of the energy drink; an increase in catalase and a decrease in SOD levels was observed after the sprint triathlon. Several studies have shown that different exercise modalities (aerobic, anaerobic, intermittent, and continuous exercises) result in different changes of SOD levels (Canale et al., 2014; Park and Kwak, 2016; Souissi et al., 2020). In our study, SOD levels showed a greater reduction in the High ED group compared to the PLA and Low ED groups. Intense exercises have been shown to increase TBARS levels; however, a reduction in TBARS levels was observed with caffeinated energy drink supplementation, in our study. This may be due to the antioxidant effect of the caffeinated energy drink. Apart from caffeine, the additional ingredients in the energy drink used in our study included elderberry and anserine. All the three ingredients can reduce oxidation stress. The use of caffeine as an antioxidant has been demonstrated in some studies (Abdel-Hady et al., 2015; Salicio et al., 2017). Caffeine can scavenge free radicals (Afify et al., 2011; León-Carmona and Galano, 2011; Costa et al., 2018; Petrucci et al., 2018), which can help decrease oxidative stress for athletes (Tiwari et al., 2014). In addition, the strong antioxidant property of elderberries is related to their phenolic compounds content, particularly flavonoids (Netzel et al., 2005; Iacopini et al., 2008; Goud and Prasad, 2020). Protective antioxidant mechanisms of anserine have also been demonstrated in some research studies (Alkhatib et al., 2020; Wu, 2020). In our study, antioxidant levels were not increased. We, therefore, infer that the caffeinated energy drink used in our study may reduce oxidative damage in athletes by scavenging free radicals.

## Conclusion

Caffeinated energy drinks consumed in the inappropriate dosage may impair the exercise performance of sprint triathletes, irrespective of oxidative damage. Therefore, individual differences may be necessary to consider when consuming caffeinated energy drink supplementation to prevent side effects due to excessive intake of caffeine or other ingredients. However, it needs more researches to confirm.

## Data Availability

The original contributions presented in the study are included in the article/supplementary material, further inquiries can be directed to the corresponding author.
